# Pitfalls in Genetic Testing for Consanguineous Pediatric Populations

**DOI:** 10.1155/2022/9393042

**Published:** 2022-05-25

**Authors:** Maha Saleh, Samantha Colaiacovo, Melanie P. Napier, Asuri N. Prasad, C. Anthony Rupar, Chitra Prasad

**Affiliations:** ^1^Division of Genetics and Metabolics, Department of Pediatrics, London Health Sciences, London, Ontario, Canada; ^2^Western University, London, Ontario, Canada; ^3^Division of Neurology, Department of Pediatrics, London Health Sciences, London, Ontario, Canada; ^4^Department of Pathology and Laboratory Medicine, London Health Sciences, London, Ontario, Canada

## Abstract

We describe the diagnostic odyssey of an eight-year-old female born to consanguineous parents. Our patient presented with global developmental delay, regression, microcephaly, spastic diplegia, and leukodystrophy confirmed on brain magnetic resonance imaging (MRI). She was found on whole exome sequencing (WES) to have dual genetic diagnoses. The first was a homozygous pathogenic HERC2 gene partial deletion of exons 43–45 that causes HERC2-related disorder. The second was a homozygous pathogenic variant (c.836 C > T, p.A279 V) in the SUMF1 gene responsible for multiple sulfatase deficiency. This case highlights some of the challenges in diagnosing consanguineous pediatric populations where standard genetic and metabolic testing may not provide answers. Our case further supports the recent American College of Medical Genetics and Genomics (ACMG) recommendation of WES as a first or second-tier test for patients with developmental delay, particularly in a population where the chances of dual diagnosis is high.

## 1. Case Report

An eight-year-old female of Iraqi descent presented to the Department of Clinical Genetics with a history of global developmental delay, regression, microcephaly, spastic diplegia, and leukodystrophy confirmed on brain magnetic resonance imaging (MRI).

The patient was the fourth child born to double first cousins. She had two older healthy siblings and a brother who died at 12 days of age following an infection. The patient had an unremarkable perinatal course and was slowly meeting her developmental milestones. At four years, she developed fever, following which she lost the ability to walk independently and showed signs of developmental regression. At that time, she had her first episode of myoclonic seizures.

**Figure 1:**
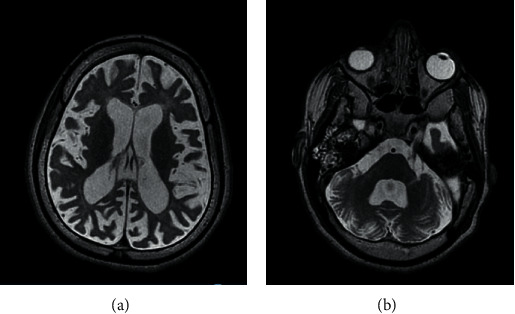
Axial T2 sequences (a) showing extensive cortical atrophy and ventriculomegaly ex-vacuo (b) at the level of the pons, showing an enlarged fourth ventricle and cerebellar cortical atrophy.

**Figure 2:**
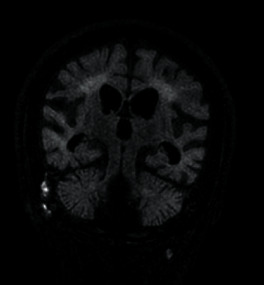
Coronal FLAIR sequence showing periventricular increased FLAIR signal consistent with white matter change, ventriculomegaly, including enlargement of the temporal horns, hippocampal, and generalized cortical and cerebellar atrophy.

At age six, the family emigrated to Canada. Within a year, the patient was wheelchair bound and had lost spontaneous speech. Ophthalmologic and hearing exams were normal. A neurological assessment identified microcephaly (<1 percentile), poor growth parameters (weight and height were at 5th percentile), and a pattern of spastic tetraparesis. MRI scan of the head showed generalized cerebral atrophy and ventriculomegaly with periventricular white matter volume loss (Figures 1 and 2). Electroencephalogram (EEG) confirmed frequent bitemporal epileptiform discharges.

Chromosomal microarray analysis identified greater than 21% absence of heterozygosity (AOH) and no copy number variants (CNV). Rett gene (MECP2) testing was normal. Metabolic screening including plasma amino acids, acylcarnitine profile, total and free carnitine, plasma lactate, ammonia, urine organic acids, and urine mucopolysaccharide screen were negative. Negative results were received from genetic testing with a 201 leukodystrophy genes panel and mitochondrial DNA panel.

At age nine, she was diagnosed with precocious puberty in addition to nonclassic, late-onset congenital adrenal hyperplasia. Cortical blindness was confirmed by an ophthalmological assessment. There were difficult to control seizures and her symptoms worsened. A request for funding of whole exome sequencing (WES) was declined on the administrative basis of the previous negative panels ordered less than three years prior.

At 12 years of age, approval for a clinical WES was granted and the results identified two diagnoses. There was a homozygous pathogenic partial HERC2 gene deletion of exons 43–45 responsible for autosomal recessive HERC2-related disorder, which is characterized by intellectual disability, gait disturbance, seizures, hypotonia, and brain anomalies. This was recently described as a cause of pediatric lethality in a consanguineous population [1, 2].

In addition, a homozygous ACMG class 1 mutation (c.836 C> T, p.A279 V) in the SUMF1 gene was identified, which is responsible for autosomal recessive multiple sulfatase deficiency (MSD). MSD is a lysosomal storage disorder that presents with developmental delay, regression, leukodystrophy, and coarse facial features [3, 4]. SUMF1 was not included in the initial leukodystrophy panel (Supplementary Material 1). The diagnosis of MSD was further confirmed biochemically by very low leukocyte arylsulfatase A activity (0.05 nmole/h/mg protein and low leukocyte arylsulfatase B activity 3 nmol//mg protein (same-day control 101 and 133). We were able to correlate the above changes to 21% AOH identified on her chromosomal microarray analysis. The AOH included 15q13.1, which harbors the HERC2 gene as well as the region 3p26.1 which carries the SUMF1 gene (Supplementary Material 2).

Her parents were identified to be carriers for those two conditions. Both parents and caregivers now have a better understanding of the cause and overall prognosis. The parents have presently accepted a referral to the palliative care team. The older siblings have also been made aware of the genetic findings and advised to seek prenatal counseling.

## 2. Discussion

This case highlights the challenges in diagnosing genetic and metabolic conditions in the growing immigrant population in Canada [5]. A position statement reviewed by the Community Pediatrics Committee includes a stepwise guide to children with developmental delay which includes formal vision and hearing testing, chromosomal microarray, and first-tier testing for treatable inborn errors of the metabolism [6].

Our patient was provided testing following these guidelines. See 21% AOH on microarray pinpoints to a high likelihood of identifying homozygous mutations for recessive disorders [7]. In addition, dual diagnoses have been commonly reported in consanguineous populations suggesting that the threshold for offering WES should be lower in patients with complex presentations in those populations [8–10].

The dual diagnoses on WES helped explain our patient's acute presentation and severe developmental regression. Our patient's pathogenic variant (c.836 C > T, p. A279 V) in the SUMF1 gene has been previously described in the literature in patients with severe infantile multiple sulfatase deficiency with regression and loss of the majority of developmental milestones by age 5 years. Prasad et al. and Miskin et al., describe clinical findings of microcephaly, neurological regression without ichthyosis, coarse facies, or organomegaly, similar to our case. The MRI changes of leukodystrophy has been described as typical finding for patients with this SUMF1 variant [11, 12].

Our patient had severe seizures that were difficult to control and was later diagnosed with cortical blindness, none of which have been previously attributed to this SUMF1 variant. However, those features were well described by Elpidorou et al., in a cohort of pediatric patients who presented with developmental delay and HERC2-related disorder. Of interest, none of the children described in that cohort had leukodystrophy on brain MRI.

This case further supports the recent American College of Medical Genetics and Genomics (ACMG) recommendation for ordering WES as a first or second-tier test for patients with developmental delay, particularly in a consanguineous population where the chances of dual diagnosis is high [13]. Despite having a unique phenotype (leukodystrophy) for which a targeted gene panel can be offered, consanguinity in a unique population can underlie other rare genetic causes not typically covered by these “extensive” and known panels.

Not many pediatricians are aware that Ministry of Health and Long-Term Care (MOHLTC) will not fund WES if an extensive panel was ordered prior. Due to public health funding regulations in Canada, WES is not funded if an extensive, disease-specific panel (>100 genes) was ordered within the prior three years.

Where possible, pediatric subspecialists should consider WES for such patients before coordinating extensive targeted panels to help shorten the diagnostic odyssey.

## Data Availability

No data were used to support this study.

## Supplementary Materials

Supplementary Materials Appendix 1. List of genes covered in the leukodystrophy panel. Appendix 2. List of AOH on chromosomal microarray analysis that includes 15q13.1 which harbors the HERC2 gene as well as 3p26.1 which carries the SUMF1 gene.

## References

[1] Elpidorou M., Best S., Poulter J. A. (2021). Novel loss-of-function mutation in HERC2 is associated with severe developmental delay and paediatric lethality. *Journal of Medical Genetics*.

[2] Harlalka G. V., Baple E. L., Cross H. (2013). Mutation of HERC2 causes developmental delay with Angelman-like features. *Journal of Medical Genetics*.

[3] Ahrens-Nicklas R., Schlotawa L., Ballabio A. (2018). Complex care of individuals with multiple sulfatase deficiency: clinical cases and consensus statement. *Molecular Genetics and Metabolism*.

[4] Schlotawa L., Adang L., De Castro M. (2019). *Multiple Sulfatase Deficiency*.

[5] Bhayana A., Bhayana B. (2018). Approach to developmental disabilities in newcomer families. *Canadian Family Physician*.

[6] Bélanger S. A., Caron J. (2018). Evaluation of the child with global developmental delay and intellectual disability. *Paediatrics and Child Health*.

[7] Kearney H. M., Kearney J. B., Conlin L. K. (2011). Diagnostic implications of excessive homozygosity detected by SNP-based microarrays: consanguinity, uniparental disomy, and recessive single-gene mutations. *Clinics in Laboratory Medicine*.

[8] Al-Dewik N., Mohd H., Al-Mureikhi M. (2019). Clinical exome sequencing in 509 Middle Eastern families with suspected Mendelian diseases: the Qatari experience. *American Journal of Medical Genetics, Part A*.

[9] Yavarna T., Al-Dewik N., Al-Mureikhi M. (2015). High diagnostic yield of clinical exome sequencing in Middle Eastern patients with Mendelian disorders. *Human Genetics*.

[10] Balci T. B., Hartley T., Xi Y. (2017). Diagnosing multiple genetic diseases in families by whole-exome sequencing. *Clinical Genetics*.

[11] Prasad C., Rupar C. A., Campbell C. (2014). Case of multiple sulfatase deficiency and ocular albinism: a diagnostic odyssey. *The Canadian Journal of Neurological Sciences*.

[12] Miskin C., Melvin J. J., Legido A., Wenger D. A., Harasink S. M., Khurana D. S. (2016). A patient with atypical multiple sulfatase deficiency. *Pediatric Neurology*.

[13] Manickam K., McClain M. R., Demmer L. A. (2021). Exome and genome sequencing for pediatric patients with congenital anomalies or intellectual disability: an evidence-based clinical guideline of the American College of Medical Genetics and Genomics (ACMG). *Genetics in Medicine*.

