# Supplementary material for: Pitfalls in Genetic Testing for Consanguineous Pediatric Populations
Source: Case Rep Genet. 2022 May 25;2022:9393042. doi: 10.1155/2022/9393042 (PMC9159873; doi:10.1155/2022/9393042)
Supplement: Supplementary Materials — Appendix 1. List of genes covered in the leukodystrophy panel. Appendix 2. List of AOH on chromosomal microarray analysis that includes 15q13.1 which harbors the HERC2 gene as well as 3p26.1 which carries the SUMF1 gene. [file 9393042.f1.zip › Appendix 1 (1).docx]

**Appendix 1- List of Genes Covered in the Leukodystrophy Panel**

| [ABAT](https://mnglabs.labcorp.com/testing?genes%5b%5d=ABAT)  [ABCA1](https://mnglabs.labcorp.com/testing?genes%5b%5d=ABCA1)  [ABCD1](https://mnglabs.labcorp.com/testing?genes%5b%5d=ABCD1)  [ACOX1](https://mnglabs.labcorp.com/testing?genes%5b%5d=ACOX1)  [ADAR](https://mnglabs.labcorp.com/testing?genes%5b%5d=ADAR)  [ADK](https://mnglabs.labcorp.com/testing?genes%5b%5d=ADK)  [AIMP1](https://mnglabs.labcorp.com/testing?genes%5b%5d=AIMP1)  [ALDH3A2](https://mnglabs.labcorp.com/testing?genes%5b%5d=ALDH3A2)  [APOA1BP](https://mnglabs.labcorp.com/testing?genes%5b%5d=APOA1BP)  [APOPT1](https://mnglabs.labcorp.com/testing?genes%5b%5d=APOPT1)  [ARCN1](https://mnglabs.labcorp.com/testing?genes%5b%5d=ARCN1)  [ARHGAP31](https://mnglabs.labcorp.com/testing?genes%5b%5d=ARHGAP31)  [ARSA](https://mnglabs.labcorp.com/testing?genes%5b%5d=ARSA)  [ASPA](https://mnglabs.labcorp.com/testing?genes%5b%5d=ASPA)  [ASXL1](https://mnglabs.labcorp.com/testing?genes%5b%5d=ASXL1)  [ASXL2](https://mnglabs.labcorp.com/testing?genes%5b%5d=ASXL2)  [AUH](https://mnglabs.labcorp.com/testing?genes%5b%5d=AUH)  [B3GALNT2](https://mnglabs.labcorp.com/testing?genes%5b%5d=B3GALNT2)  [BCAP31](https://mnglabs.labcorp.com/testing?genes%5b%5d=BCAP31)  [BCL11B](https://mnglabs.labcorp.com/testing?genes%5b%5d=BCL11B)  [BRAT1](https://mnglabs.labcorp.com/testing?genes%5b%5d=BRAT1)  [C11orf73](https://mnglabs.labcorp.com/testing?genes%5b%5d=C11orf73)  [ISCA2](https://mnglabs.labcorp.com/testing?genes%5b%5d=ISCA2)  [ITPA](https://mnglabs.labcorp.com/testing?genes%5b%5d=ITPA)  [JAM3](https://mnglabs.labcorp.com/testing?genes%5b%5d=JAM3)  [KCNJ10](https://mnglabs.labcorp.com/testing?genes%5b%5d=KCNJ10)  [KCNT1](https://mnglabs.labcorp.com/testing?genes%5b%5d=KCNT1)  [KDM1A](https://mnglabs.labcorp.com/testing?genes%5b%5d=KDM1A)  [KIAA0586](https://mnglabs.labcorp.com/testing?genes%5b%5d=KIAA0586)  [KLHL15](https://mnglabs.labcorp.com/testing?genes%5b%5d=KLHL15)  [L2HGDH](https://mnglabs.labcorp.com/testing?genes%5b%5d=L2HGDH)  [LAGE3](https://mnglabs.labcorp.com/testing?genes%5b%5d=LAGE3)  [LAMA1](https://mnglabs.labcorp.com/testing?genes%5b%5d=LAMA1)  [LIPT2](https://mnglabs.labcorp.com/testing?genes%5b%5d=LIPT2)  [LMNB1](https://mnglabs.labcorp.com/testing?genes%5b%5d=LMNB1)  [LONP1](https://mnglabs.labcorp.com/testing?genes%5b%5d=LONP1)  [MAG](https://mnglabs.labcorp.com/testing?genes%5b%5d=MAG)  [MARS2](https://mnglabs.labcorp.com/testing?genes%5b%5d=MARS2)  [MED17](https://mnglabs.labcorp.com/testing?genes%5b%5d=MED17)  [MLC1](https://mnglabs.labcorp.com/testing?genes%5b%5d=MLC1)  [MLYCD](https://mnglabs.labcorp.com/testing?genes%5b%5d=MLYCD)  [MPV17](https://mnglabs.labcorp.com/testing?genes%5b%5d=MPV17)  [MRPS22](https://mnglabs.labcorp.com/testing?genes%5b%5d=MRPS22)  [MTFMT](https://mnglabs.labcorp.com/testing?genes%5b%5d=MTFMT)  [MTOR](https://mnglabs.labcorp.com/testing?genes%5b%5d=MTOR)  [PPP1R15B](https://mnglabs.labcorp.com/testing?genes%5b%5d=PPP1R15B)  [PPP2R1A](https://mnglabs.labcorp.com/testing?genes%5b%5d=PPP2R1A)  [PPP3CA](https://mnglabs.labcorp.com/testing?genes%5b%5d=PPP3CA)  [PRKDC](https://mnglabs.labcorp.com/testing?genes%5b%5d=PRKDC)  [PRPS1](https://mnglabs.labcorp.com/testing?genes%5b%5d=PRPS1)  [PSAP](https://mnglabs.labcorp.com/testing?genes%5b%5d=PSAP)  [PUS3](https://mnglabs.labcorp.com/testing?genes%5b%5d=PUS3)  [PYCR2](https://mnglabs.labcorp.com/testing?genes%5b%5d=PYCR2)  [QARS](https://mnglabs.labcorp.com/testing?genes%5b%5d=QARS)  [RAC1](https://mnglabs.labcorp.com/testing?genes%5b%5d=RAC1)  [RBM8A](https://mnglabs.labcorp.com/testing?genes%5b%5d=RBM8A)  [RERE](https://mnglabs.labcorp.com/testing?genes%5b%5d=RERE)  [RNASEH2A](https://mnglabs.labcorp.com/testing?genes%5b%5d=RNASEH2A)  [RNASEH2B](https://mnglabs.labcorp.com/testing?genes%5b%5d=RNASEH2B)  [RNASEH2C](https://mnglabs.labcorp.com/testing?genes%5b%5d=RNASEH2C)  [RNASET2](https://mnglabs.labcorp.com/testing?genes%5b%5d=RNASET2)  [RPIA](https://mnglabs.labcorp.com/testing?genes%5b%5d=RPIA)  [SAMD9](https://mnglabs.labcorp.com/testing?genes%5b%5d=SAMD9)  [SAMHD1](https://mnglabs.labcorp.com/testing?genes%5b%5d=SAMHD1)  [SCP2](https://mnglabs.labcorp.com/testing?genes%5b%5d=SCP2)  [SDHA](https://mnglabs.labcorp.com/testing?genes%5b%5d=SDHA)  [SDHAF1](https://mnglabs.labcorp.com/testing?genes%5b%5d=SDHAF1)  [SDHB](https://mnglabs.labcorp.com/testing?genes%5b%5d=SDHB) | [C2CD3](https://mnglabs.labcorp.com/testing?genes%5b%5d=C2CD3)  [CCDC88A](https://mnglabs.labcorp.com/testing?genes%5b%5d=CCDC88A)  [CIC](https://mnglabs.labcorp.com/testing?genes%5b%5d=CIC)  [CLCN2](https://mnglabs.labcorp.com/testing?genes%5b%5d=CLCN2)  [CLN6](https://mnglabs.labcorp.com/testing?genes%5b%5d=CLN6)  [CNNM2](https://mnglabs.labcorp.com/testing?genes%5b%5d=CNNM2)  [COL4A1](https://mnglabs.labcorp.com/testing?genes%5b%5d=COL4A1)  [COL4A2](https://mnglabs.labcorp.com/testing?genes%5b%5d=COL4A2)  [COX15](https://mnglabs.labcorp.com/testing?genes%5b%5d=COX15)  [CRIPT](https://mnglabs.labcorp.com/testing?genes%5b%5d=CRIPT)  [CRLF1](https://mnglabs.labcorp.com/testing?genes%5b%5d=CRLF1)  [CSF1R](https://mnglabs.labcorp.com/testing?genes%5b%5d=CSF1R)  [CTC1](https://mnglabs.labcorp.com/testing?genes%5b%5d=CTC1)  [CTNS](https://mnglabs.labcorp.com/testing?genes%5b%5d=CTNS)  [CYB5R3](https://mnglabs.labcorp.com/testing?genes%5b%5d=CYB5R3)  [CYP27A1](https://mnglabs.labcorp.com/testing?genes%5b%5d=CYP27A1)  [DAG1](https://mnglabs.labcorp.com/testing?genes%5b%5d=DAG1)  [DARS](https://mnglabs.labcorp.com/testing?genes%5b%5d=DARS)  [DARS2](https://mnglabs.labcorp.com/testing?genes%5b%5d=DARS2)  [DMXL2](https://mnglabs.labcorp.com/testing?genes%5b%5d=DMXL2)  [DONSON](https://mnglabs.labcorp.com/testing?genes%5b%5d=DONSON)  [EARS2](https://mnglabs.labcorp.com/testing?genes%5b%5d=EARS2)  [NADK2](https://mnglabs.labcorp.com/testing?genes%5b%5d=NADK2)  [NDUFA1](https://mnglabs.labcorp.com/testing?genes%5b%5d=NDUFA1)  [NDUFA10](https://mnglabs.labcorp.com/testing?genes%5b%5d=NDUFA10)  [NDUFA11](https://mnglabs.labcorp.com/testing?genes%5b%5d=NDUFA11)  [NDUFA12](https://mnglabs.labcorp.com/testing?genes%5b%5d=NDUFA12)  [NDUFA2](https://mnglabs.labcorp.com/testing?genes%5b%5d=NDUFA2)  [NDUFA9](https://mnglabs.labcorp.com/testing?genes%5b%5d=NDUFA9)  [NDUFAF1](https://mnglabs.labcorp.com/testing?genes%5b%5d=NDUFAF1)  [NDUFAF2](https://mnglabs.labcorp.com/testing?genes%5b%5d=NDUFAF2)  [NDUFAF3](https://mnglabs.labcorp.com/testing?genes%5b%5d=NDUFAF3)  [NDUFAF4](https://mnglabs.labcorp.com/testing?genes%5b%5d=NDUFAF4)  [NDUFAF5](https://mnglabs.labcorp.com/testing?genes%5b%5d=NDUFAF5)  [NDUFAF6](https://mnglabs.labcorp.com/testing?genes%5b%5d=NDUFAF6)  [NDUFB3](https://mnglabs.labcorp.com/testing?genes%5b%5d=NDUFB3)  [NDUFS1](https://mnglabs.labcorp.com/testing?genes%5b%5d=NDUFS1)  [NDUFS2](https://mnglabs.labcorp.com/testing?genes%5b%5d=NDUFS2)  [NDUFS3](https://mnglabs.labcorp.com/testing?genes%5b%5d=NDUFS3)  [NDUFS4](https://mnglabs.labcorp.com/testing?genes%5b%5d=NDUFS4)  [NDUFS6](https://mnglabs.labcorp.com/testing?genes%5b%5d=NDUFS6)  [NDUFS7](https://mnglabs.labcorp.com/testing?genes%5b%5d=NDUFS7)  [NDUFS8](https://mnglabs.labcorp.com/testing?genes%5b%5d=NDUFS8)  [PTEN](https://mnglabs.labcorp.com/testing?genes%5b%5d=PTEN)  [SLC12A5](https://mnglabs.labcorp.com/testing?genes%5b%5d=SLC12A5)  [SLC16A2](https://mnglabs.labcorp.com/testing?genes%5b%5d=SLC16A2)  [SLC1A2](https://mnglabs.labcorp.com/testing?genes%5b%5d=SLC1A2)  [SLC1A4](https://mnglabs.labcorp.com/testing?genes%5b%5d=SLC1A4)  [SLC25A12](https://mnglabs.labcorp.com/testing?genes%5b%5d=SLC25A12)  [SLC25A15](https://mnglabs.labcorp.com/testing?genes%5b%5d=SLC25A15)  [SLC6A9](https://mnglabs.labcorp.com/testing?genes%5b%5d=SLC6A9)  [SMG9](https://mnglabs.labcorp.com/testing?genes%5b%5d=SMG9)  [SNIP1](https://mnglabs.labcorp.com/testing?genes%5b%5d=SNIP1)  [SNORD118](https://mnglabs.labcorp.com/testing?genes%5b%5d=SNORD118)  [SNRPB](https://mnglabs.labcorp.com/testing?genes%5b%5d=SNRPB)  [SON](https://mnglabs.labcorp.com/testing?genes%5b%5d=SON)  [SOX10](https://mnglabs.labcorp.com/testing?genes%5b%5d=SOX10)  [SOX2](https://mnglabs.labcorp.com/testing?genes%5b%5d=SOX2)  [SPATA5](https://mnglabs.labcorp.com/testing?genes%5b%5d=SPATA5)  [SPG20](https://mnglabs.labcorp.com/testing?genes%5b%5d=SPG20)  [STAMBP](https://mnglabs.labcorp.com/testing?genes%5b%5d=STAMBP)  [STXBP1](https://mnglabs.labcorp.com/testing?genes%5b%5d=STXBP1)  [TACO1](https://mnglabs.labcorp.com/testing?genes%5b%5d=TACO1)  [TAF2](https://mnglabs.labcorp.com/testing?genes%5b%5d=TAF2)  [TARS2](https://mnglabs.labcorp.com/testing?genes%5b%5d=TARS2)  [TBCD](https://mnglabs.labcorp.com/testing?genes%5b%5d=TBCD)  [TBCE](https://mnglabs.labcorp.com/testing?genes%5b%5d=TBCE) | [EIF2B1](https://mnglabs.labcorp.com/testing?genes%5b%5d=EIF2B1)  [EIF2B2](https://mnglabs.labcorp.com/testing?genes%5b%5d=EIF2B2)  [EIF2B3](https://mnglabs.labcorp.com/testing?genes%5b%5d=EIF2B3)  [EIF2B4](https://mnglabs.labcorp.com/testing?genes%5b%5d=EIF2B4)  [EIF2B5](https://mnglabs.labcorp.com/testing?genes%5b%5d=EIF2B5)  [EXOSC8](https://mnglabs.labcorp.com/testing?genes%5b%5d=EXOSC8)  [FA2H](https://mnglabs.labcorp.com/testing?genes%5b%5d=FA2H)  [FAM126A](https://mnglabs.labcorp.com/testing?genes%5b%5d=FAM126A)  [FBXL4](https://mnglabs.labcorp.com/testing?genes%5b%5d=FBXL4)  [FKRP](https://mnglabs.labcorp.com/testing?genes%5b%5d=FKRP)  [GALC](https://mnglabs.labcorp.com/testing?genes%5b%5d=GALC)  [GFAP](https://mnglabs.labcorp.com/testing?genes%5b%5d=GFAP)  [GJC2](https://mnglabs.labcorp.com/testing?genes%5b%5d=GJC2)  [GLRX5](https://mnglabs.labcorp.com/testing?genes%5b%5d=GLRX5)  [GLYCTK](https://mnglabs.labcorp.com/testing?genes%5b%5d=GLYCTK)  [GMNN](https://mnglabs.labcorp.com/testing?genes%5b%5d=GMNN)  [GPR56](https://mnglabs.labcorp.com/testing?genes%5b%5d=GPR56)  [HACE1](https://mnglabs.labcorp.com/testing?genes%5b%5d=HACE1)  [HEPACAM](https://mnglabs.labcorp.com/testing?genes%5b%5d=HEPACAM)  [HNRNPU](https://mnglabs.labcorp.com/testing?genes%5b%5d=HNRNPU)  [HSPD1](https://mnglabs.labcorp.com/testing?genes%5b%5d=HSPD1)  [HTRA1](https://mnglabs.labcorp.com/testing?genes%5b%5d=HTRA1)  [MTTP](https://mnglabs.labcorp.com/testing?genes%5b%5d=MTTP)  [NACC1](https://mnglabs.labcorp.com/testing?genes%5b%5d=NACC1)  [NDUFV1](https://mnglabs.labcorp.com/testing?genes%5b%5d=NDUFV1)  [NDUFV2](https://mnglabs.labcorp.com/testing?genes%5b%5d=NDUFV2)  [NEK1](https://mnglabs.labcorp.com/testing?genes%5b%5d=NEK1)  [NOTCH3](https://mnglabs.labcorp.com/testing?genes%5b%5d=NOTCH3)  [NUBPL](https://mnglabs.labcorp.com/testing?genes%5b%5d=NUBPL)  [OBFC1](https://mnglabs.labcorp.com/testing?genes%5b%5d=OBFC1)  [OMG](https://mnglabs.labcorp.com/testing?genes%5b%5d=OMG)  [OSGEP](https://mnglabs.labcorp.com/testing?genes%5b%5d=OSGEP)  [PAH](https://mnglabs.labcorp.com/testing?genes%5b%5d=PAH)  [PC](https://mnglabs.labcorp.com/testing?genes%5b%5d=PC)  [PDCD1](https://mnglabs.labcorp.com/testing?genes%5b%5d=PDCD1)  [PET100](https://mnglabs.labcorp.com/testing?genes%5b%5d=PET100)  [PHGDH](https://mnglabs.labcorp.com/testing?genes%5b%5d=PHGDH)  [PIGP](https://mnglabs.labcorp.com/testing?genes%5b%5d=PIGP)  [PLEKHG2](https://mnglabs.labcorp.com/testing?genes%5b%5d=PLEKHG2)  [PLP1](https://mnglabs.labcorp.com/testing?genes%5b%5d=PLP1)  [POLG](https://mnglabs.labcorp.com/testing?genes%5b%5d=POLG)  [POLR1C](https://mnglabs.labcorp.com/testing?genes%5b%5d=POLR1C)  [POLR3A](https://mnglabs.labcorp.com/testing?genes%5b%5d=POLR3A)  [POLR3B](https://mnglabs.labcorp.com/testing?genes%5b%5d=POLR3B)  [TBCK](https://mnglabs.labcorp.com/testing?genes%5b%5d=TBCK)  [TIMMDC1](https://mnglabs.labcorp.com/testing?genes%5b%5d=TIMMDC1)  [TM4SF20](https://mnglabs.labcorp.com/testing?genes%5b%5d=TM4SF20)  [TMEM126B](https://mnglabs.labcorp.com/testing?genes%5b%5d=TMEM126B)  [TMTC3](https://mnglabs.labcorp.com/testing?genes%5b%5d=TMTC3)  [TP53RK](https://mnglabs.labcorp.com/testing?genes%5b%5d=TP53RK)  [TRAPPC11](https://mnglabs.labcorp.com/testing?genes%5b%5d=TRAPPC11)  [TREM2](https://mnglabs.labcorp.com/testing?genes%5b%5d=TREM2)  [TREX1](https://mnglabs.labcorp.com/testing?genes%5b%5d=TREX1)  [TUBB4A](https://mnglabs.labcorp.com/testing?genes%5b%5d=TUBB4A)  [TUFM](https://mnglabs.labcorp.com/testing?genes%5b%5d=TUFM)  [TXN2](https://mnglabs.labcorp.com/testing?genes%5b%5d=TXN2)  [TYMP](https://mnglabs.labcorp.com/testing?genes%5b%5d=TYMP)  [TYROBP](https://mnglabs.labcorp.com/testing?genes%5b%5d=TYROBP)  [UBTF](https://mnglabs.labcorp.com/testing?genes%5b%5d=UBTF)  [UPB1](https://mnglabs.labcorp.com/testing?genes%5b%5d=UPB1)  [VPS33A](https://mnglabs.labcorp.com/testing?genes%5b%5d=VPS33A)  [VARS2](https://mnglabs.labcorp.com/testing?genes%5b%5d=VARS2)  [VPS11](https://mnglabs.labcorp.com/testing?genes%5b%5d=VPS11)  [WDR73](https://mnglabs.labcorp.com/testing?genes%5b%5d=WDR73)  [YME1L1](https://mnglabs.labcorp.com/testing?genes%5b%5d=YME1L1)  [ZNHIT3](https://mnglabs.labcorp.com/testing?genes%5b%5d=ZNHIT3) |
| --- | --- | --- |
