# Supplementary material for: Pitfalls in Genetic Testing for Consanguineous Pediatric Populations
Source: Case Rep Genet. 2022 May 25;2022:9393042. doi: 10.1155/2022/9393042 (PMC9159873; doi:10.1155/2022/9393042)
Supplement: Supplementary Materials — Appendix 1. List of genes covered in the leukodystrophy panel. Appendix 2. List of AOH on chromosomal microarray analysis that includes 15q13.1 which harbors the HERC2 gene as well as 3p26.1 which carries the SUMF1 gene. [file 9393042.f1.zip › Appendix 1 (1).pdf]

Appendix 1- List of Genes Covered in the Leukodystrophy Panel

ABAT	C2CD3	EIF2B1
ABCA1	CCDC88A	EIF2B2
ABCD1	CIC	EIF2B3
ACOX1	CLCN2	EIF2B4
ADAR	CLN6	EIF2B5
ADK	CNNM2	EXOSC8
AIMP1	COL4A1	FA2H
ALDH3A2	COL4A2	FAM126A
APOA1BP	COX15	FBXL4
APOPT1	CRIPT	FKRP
ARCN1	CRLF1	GALC
ARHGAP31	CSF1R	GFAP
ARSA	CTC1	GJC2
ASPA	CTNS	GLRX5
ASXL1	CYB5R3	GLYCTK
ASXL2	CYP27A1	GMNN
AUH	DAG1	GPR56
B3GALNT2	DARS	HACE1
BCAP31	DARS2	HEPACAM
BCL11B	DMXL2	HNRNPU
BRAT1	DONSON	HSPD1
C11orf73	EARS2	HTRA1

ISCA2	NADK2	MTTP
ITPA	NDUFA1	NACC1
JAM3	NDUFA10	NDUFV1
KCNJ10	NDUFA11	NDUFV2
KCNT1	NDUFA12	NEK1
KDM1A	NDUFA2	NOTCH3
KIAA0586	NDUFA9	NUBPL
KLHL15	NDUFAF1	OBFC1
L2HGDH	NDUFAF2	OMG
LAGE3	NDUFAF3	OSGEP
LAMA1	NDUFAF4	PAH
LIPT2	NDUFAF5	PC
LMNB1	NDUFAF6	PDCD1
LONP1	NDUFB3	PET100
MAG	NDUFS1	PHGDH
MARS2	NDUFS2	PIGP
MED17	NDUFS3	PLEKHG2
MLC1	NDUFS4	PLP1
MLYCD	NDUFS6	POLG
MPV17	NDUFS7	POLR1C
MRPS22	NDUFS8	POLR3A
MTFMT	PTEN	POLR3B
MTOR		

PPP1R15B	SLC12A5	TBCK
PPP2R1A	SLC16A2	TIMMDC1
PPP3CA	SLC1A2	TM4SF20
PRKDC	SLC1A4	TMEM126B
PRPS1	SLC25A12	TMTC3
PSAP	SLC25A15	TP53RK
PUS3	SLC6A9	TRAPPC11
PYCR2	SMG9	TREM2
QARS	SNIP1	TREX1
RAC1	SNORD118	TUBB4A
RBM8A	SNRPB	TUFM
RERE	SON	TXN2
RNASEH2A	SOX10	TYMP
RNASEH2B	SOX2	TYROBP
RNASEH2C	SPATA5	UBTF
RNASET2	SPG20	UPB1
RPIA	STAMBP	VPS33A
SAMD9	STXBP1	VARS2
SAMHD1	TACO1	VPS11
SCP2	TAF2	WDR73
SDHA	TARS2	YME1L1
SDHAF1	TBCD	ZNHIT3
SDHB	TBCE	
