# Supplementary material for: Pitfalls in Genetic Testing for Consanguineous Pediatric Populations
Source: Case Rep Genet. 2022 May 25;2022:9393042. doi: 10.1155/2022/9393042 (PMC9159873; doi:10.1155/2022/9393042)
Supplement: Supplementary Materials — Appendix 1. List of genes covered in the leukodystrophy panel. Appendix 2. List of AOH on chromosomal microarray analysis that includes 15q13.1 which harbors the HERC2 gene as well as 3p26.1 which carries the SUMF1 gene. [file 9393042.f1.zip › Appenidx 2 List of AOH Regions (1).docx]

| ***List of AOH Regions****  arr[hg19] 1p36.22p34.3(12,153,412-37,971,212)x2 hmz  arr[hg19] 1q32.3q41(213,932,820-220,850,116)x2 hmz  arr[hg19] 3p26.3p24.2(62,613-24,745,867)x2 hmz  arr[hg19] 3p22.1p14.2(41,065,412-62,604,010)x2 hmz  arr[hg19] 3p13p11.1(72,179,437-90,485,635)x2 hmz  arr[hg19] 3q11.1q11.2(93,536,053-97,806,175)x2 hmz  arr[hg19] 3q13.31q23(117,056,382-139,042,283)x2 hmz  arr[hg19] 4q25q27(109,981,763-121,791,812)x2 hmz  arr[hg19] 4q28.3q32.3(135,321,740-168,636,895)x2 hmz  arr[hg19] 4q33q34.1(170,670,945-174,458,491)x2 hmz  arr[hg19] 5p15.33p13.3(3,394,893-31,765,790)x2 hmz  arr[hg19] 6p22.3p21.32(24,799,242-32,968,412)x2 hmz  arr[hg19] 6q11.1q12(61,968,745-65,915,022)x2 hmz  arr[hg19] 6q15q16.1(92,840,727-95,841,494)x2 hmz  arr[hg19] 7p22.1p15.3(5,832,632-22,159,571)x2 hmz  arr[hg19] 7q35q36.3(147,752,410-155,300,047)x2 hmz  arr[hg19] 8p21.3p11.1(20,811,507-43,778,914)x2 hmz  arr[hg19] 8q11.1q22.2(46,913,605-101,476,085)x2 hmz  arr[hg19] 8q23.1q24.11(106,778,063-119,085,207)x2 hmz  arr[hg19] 9p21.3p13.2(23,197,357-37,694,070)x2 hmz  arr[hg19] 9q21.33q31.1(90,276,665-105,973,033)x2 hmz  arr[hg19] 9q33.3q34.13(129,505,767-134,676,615)x2 hmz  arr[hg19] 10q11.21q21.1(43,947,626-54,061,438)x2 hmz  arr[hg19] 10q23.1q26.2(82,611,441-128,339,955)x2 hmz  arr[hg19] 12p13.31p12.1(7,420,324-22,956,137)x2 hmz  arr[hg19] 12q14.3q23.2(67,288,718-103,045,339)x2 hmz  arr[hg19] 12q23.3q24.32(106,332,287-126,307,790)x2 hmz  arr[hg19] 13q12.11q12.12(21,581,122-24,668,520)x2 hmz  arr[hg19] 13q12.3q14.11(31,608,474-43,477,731)x2 hmz  arr[hg19] 14q32.31q32.33(101,533,924-107,285,437)x2 hmz  arr[hg19] 15q11.2q13.1(22,752,398-29,440,491)x2 hmz  arr[hg19] 15q21.1q21.3(47,489,020-57,863,724)x2 hmz  arr[hg19] 15q26.2q26.3(96,112,868-102,104,049)x2 hmz  arr[hg19] 16p11.2p11.1(31,873,872-35,220,544)x2 hmz**  arr[hg19] 17q22q25.3(51,439,619-77,016,317)x2 hmz  arr[hg19] 18p11.31p11.21(3,502,812-15,143,714)x2 hmz  arr[hg19] 20q13.33(59,356,369-62,913,996)x2 hmz  arr[hg19] 22q11.21q12.1(18,640,729-26,629,095)x2 hmz  arr[hg19] Xp11.21p11.1(55,289,719-58,337,890)x2 hmz**  arr[hg19] Xq11.1q12(61,932,503-65,823,606)x2 hmz**  arr[hg19] Xq23q25(109,994,232-121,854,516)x2 hmz |
| --- |

**Appendix 2**- List of AOH on chromosomal microarray analysis that includes 15q13.1 which harbors the *HERC2* gene as well as 3p26.1 which carries the *SUMF1* gene (highlighted below).
